# Closed Reduction and Percutaneous Pinning in the Treatment of Humeral Distal Metaphyseal-Diaphyseal Junction Fractures in Children: A Technique Note and Preliminary Results

**DOI:** 10.3389/fped.2021.670164

**Published:** 2021-06-17

**Authors:** Hai Zhou, Ge Zhang, Ming Li, Xing Liu, Xiangyang Qu, Yujiang Cao, Liuqi Weng, Yuan Zhang

**Affiliations:** ^1^Department of Orthopaedics, Children's Hospital of Chongqing Medical University, Ministry of Education Key Laboratory of Child Development and Disorders, National Clinical Research Center for Child Health and Disorders, Chongqing Medical University, Chongqing, China; ^2^Chongqing Key Laboratory of Pediatrics, Chongqing, China

**Keywords:** humeral, distal metaphyseal-diaphyseal junction, fracture, closed reduction and percutaneous pinning, children

## Abstract

**Objective:** The metaphyseal-diaphyseal junction (MDJ) fracture is an uncommon but problematic type of fracture occurring at the distal humerus in children. Closed reduction and fixation are challenging and may not be possible with the conventional reduction maneuver utilized in supracondylar fractures. The purpose of this study was to evaluate a novel closed reduction and percutaneous pinning (CRPP) technique for the treatment of these fractures.

**Methods:** We retrospectively evaluated 14 children (8 boys and 6 girls) who underwent closed reduction and percutaneous fixation for the treatment of MDJ fractures. Six children who underwent treatment with a novel CRPP technique were enrolled as Group A. Eight children underwent the conventional reduction maneuver utilized in supracondylar fracture and were enrolled as Group B. Clinical and radiographic outcomes in the two groups were then compared.

**Results:** In Group A, all six MDJ fractures were treated successfully with the novel CRPP technique without the need for open procedures or re-operation. No complications such as pin-site infection or iatrogenic nerve injury were found in this group. In group B, five of the eight fractures were treated successfully with the conventional CRPP technique; three fractures needed open reduction, and one of them had further surgery because of the loss of fixation. Children with successful CRPP in each group were included to compare the efficacy of the novel CRPP technique. The average duration of the surgery in Group A was significantly shorter than that in Group B (*p* < 0.001). At last follow-up, both groups obtained satisfactory clinical and radiographic outcomes.

**Conclusion:** MDJ fractures can be reduced successfully and fixed stably via a novel CRPP technique, and laborious and frustrating attempts at closed reduction and further open reduction can be avoided.

## Introduction

The humeral supracondylar fracture is the most common elbow fracture in children (1), accounting for 55–75% of elbow fractures in children (2). At present, a normalized treatment algorithm for the fracture has been established (3–5). Briefly, it recommends non-surgical immobilization for non-displaced fractures and closed reduction with percutaneous pinning for displaced fractures (6).

A specific variant of humeral supracondylar fracture has been reported in which the fracture line crosses just proximal to the olecranon fossa. This kind of atypical supracondylar fracture has been defined as a distal humeral metaphyseal-diaphyseal junction (MDJ) fracture (7, 8). The MDJ fracture in children is rare, and it accounts for only 3.3% of displaced fractures at the distal humerus (9, 10). MDJ fractures are problematic to treat because of their instability and tendency to develop post-operative complications (8, 9, 11). The main reason for these difficulties is that the fracture line of MDJ fracture is above the olecranon fossa, making it difficult to obtain adequate stability during manual reduction because the cross-sectional area is much smaller than that in the supracondylar region, and the pins tend to cross through the higher fracture site with a route nearly parallel to the humeral axis, which may lead to the decrease of fixation stability (12). Unfortunately, in most circumstances, the characteristics of MDJ fractures are not currently recognized by all colleagues, and some MDJ fractures are still being treated with the same modality as typical supracondylar humerus fractures, which has caused more complications such as higher incidence of need for open procedures and loss of fixation (9, 13).

The paramount goal of this study was to introduce a novel surgical technique in treating distal humeral MDJ fractures in children and further evaluate the preliminary clinical and radiographic outcomes of this modality.

## Patients and Methods

### Study Participants

This retrospective single-center observational study was approved by the Institutional Review Board of our institution. The definition of MDJ fracture is a displaced distal humeral fracture where the fracture line passes proximal to the olecranon fossa but involves the flaring part of the distal humerus (from the area where the consistent width of the humerus starts changing up to the tip of the olecranon fossa) (14, 15). The study included 14 children with a diagnosis of displaced MDJ fracture at the distal humerus between January 2016 and December 2019 at our single tertiary hospital. From March 2018, we started to utilize a novel closed reduction and percutaneous pinning (CRPP) technique for the treatment of MDJ fractures. Since then, children with the diagnosis of MDJ have been treated with the novel technique, and a total of six children were included in this study (Group A). Another eight children who had undergone operative procedures according to conventional methods for typical supracondylar fractures before March 2018 were enrolled as a control group (Group B). Medical records were reviewed to identify demographic and clinical data including sex, age, injury side, type of the fracture (oblique, transverse, or comminuted), and neurovascular status.

### Surgical Techniques

**Figure 1:**
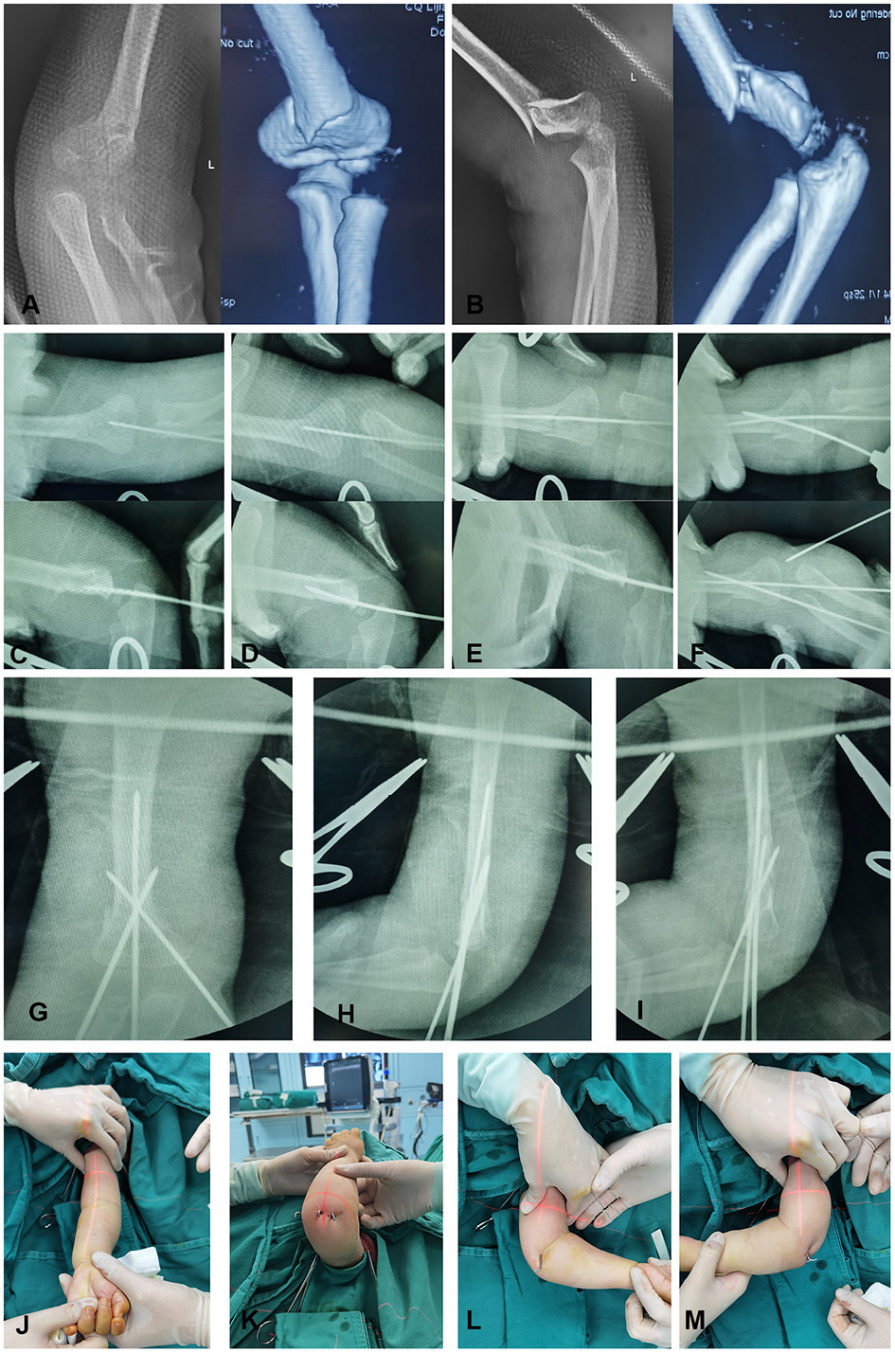
A 2-year-old girl with displaced MDJ fracture of her left elbow. **(A)** The anteroposterior (AP) radiograph and coronal 3D-CT image of the MDJ fracture; **(B)** the lateral radiograph and sagittal 3D-CT image of the fracture; **(C)** the AP and lateral radiographs when inserting a pin into the distal fracture end; **(D)** the AP and lateral radiographs where the intramedullary pin crosses the fracture line; **(E)** the AP and lateral radiographs when driving the pin through the fracture line and into the intramedullary canal; **(F)** the AP radiographs of cross-pin placement; the AP **(G)**, lateral **(H)**, and oblique **(I)** radiographs after cross-pin configuration; **(J–M)** the post-operative appearance of the elbow after this novel, minimally invasive procedure.

The surgery was performed under general anesthesia with the children in the supine position. Children in Group A underwent the operation based on a novel technique in which maneuvers were reduced and pin placements were different from those usually employed in treating supracondylar humeral fractures. After confirming displacement of the MDJ humeral fracture under intraoperative C-arm fluoroscopy, a Kirschner wire (K-wire) (SanatMetal Ltd., Eger, Hungary) with diameters of 1.8–2.0 mm was first inserted into the distal fracture fragment axially under fluoroscopic guidance. Next, the injured extremity was placed in axial traction, and the coronal alignment, overlap, and mediolateral translation were corrected under anteroposterior (AP) fluoroscopy. Following the correction of frontal displacement, the elbow was held by the assistant to maintain the reduction, with no movement of the child's arm, and the C-arm was rotated by 90° to obtain a lateral image of the elbow. Then, the sagittal angulation and/or translation was corrected by flexing the elbow as well as by anterior or posterior translation if needed. At this stage, we ensured that the maneuver was minimal while monitoring by fluoroscopy, because the periosteum at the MDJ site is vulnerable to being torn by any excessive manipulation, and overflexion of the affected elbow may lead to the destruction of the integrity of the periosteum. Subsequently, the pre-pinning K-wire was placed across the fracture site and into the proximal marrow cavity under the elbow held stable by the assistant. For some cases, rotation was needed for correction by selectively pronating or supinating the forearm. Once the reduction was reassessed under AP, oblique, and lateral fluoroscopy, two K-wires of appropriate diameter were inserted percutaneously the from lateral condyle and medial epicondyle in a crossed-pin configuration (Figure 1). Flexion, extension, and rotation of the elbow were conducted to ensure the stability of the fracture after fixation. If the reduction was judged to be unacceptable, an open reduction procedure was employed.

Children in Group B underwent the surgery according to the technique typically utilized in supracondylar humerus fractures as described by Skaggs (16). Briefly, patients were in the supine position, and coronal and sagittal displacements were corrected under intraoperative C-arm fluoroscopy. The maneuver was similar to that in Group A, but no intramedullary K-wire was placed initially. After the reductions were confirmed under intraoperative radiography, two or three K-wires were inserted percutaneously in a crossed-pin configuration. Similarly, if the reduction or the fixation was unacceptable, an open procedure was employed.

**Figure 2:**
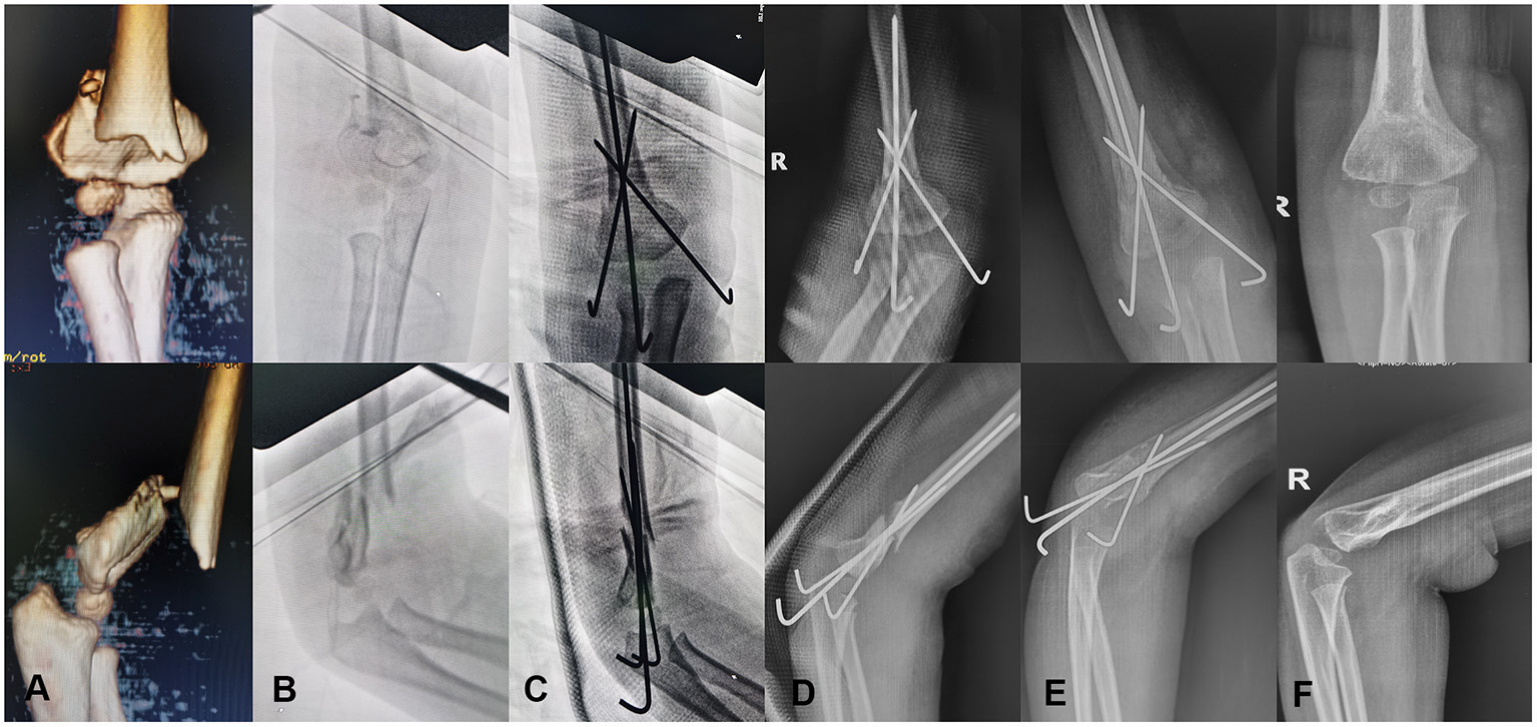
A 4-year-old boy with displaced MDJ fracture of his right elbow. **(A)** The coronal and sagittal 3D-CT images of the fracture; **(B)** the AP and lateral radiographs of the fracture before intraoperative reduction; **(C)** the AP and lateral radiographs after the utilization of the novel CRPP procedure intraoperatively; **(D)** the AP and lateral radiographs at 1 day after surgery; **(E)** the AP and lateral radiographs at the time of pin removal 4 weeks post-operatively; **(F)** the AP and lateral radiographs at 12 months post-operative follow-up.

Thereafter, the pin tails were then cut and bent and the affected arm was placed in a long arm cast to immobilize the fracture until the removal of the internal fixation pins. The K-wires were removed in the outpatient clinic when fracture healing was documented on two views (4–6 weeks post-operatively). All children had at least 1 year of follow-up (Figure 2).

### Outcome Evaluation

The following were recorded in all patients: need for open reduction, surgery duration, the number and configuration of K-wires used for fixation, pin-site infection, need for re-operation, and complications. At last follow-up, children were excluded from the study if they required an open reduction or re-operation, and children with MDJ who underwent successful CRPP were included for further analysis. Clinical outcomes including the range of motion (ROM) and the clinical carrying angle of the injured and contralateral elbows were measured with a goniometer. The clinical results were evaluated using the system described by Flynn (17). The radiographic outcomes including Baumann's angles and lateral humeral-capitellar angles immediately after operation, at the time of pin removal, and at latest follow-up were assessed on digital images by the measuring tool of the radiographic system.

### Statistical Analysis

All variables were analyzed with SPSS 22.0 statistical software; continuous data were indicated by *X* ± SD, and the Student ANOVA analysis was used for the comparison of continuous variables. The chi-square test was used for categorical variables. The level of statistical significance was determined at *p* < 0.05.

## Results

The demographic information on the patients is shown in Table 1. No nerve palsy or vascular injury at initial evaluation was present in either group. All the six fractures in Group A were successfully reduced by closed manipulation under fluoroscopic control and stabilized using one intramedullary K-wire insertion accompanied by two other K-wires in a crossed configuration. None of the fractures was treated with surgical open reduction. The average duration of the operation was 41.67 ± 7.53 min (range, 35–55 min). There was no complication such as pin-site infection, loss of fixation, iatrogenic nerve injury, or need for further surgery. In Group B, all the fractures were initially treated by closed reduction, and five of the eight had an acceptable reduction and were stabilized with percutaneous pinning. Three other children needed open reduction because of difficulty in pinning or inadequate fixation. Overall, the incidence of the need for an open procedure in Group B (37.5%, 3/8) was higher than that in Group A (0%, 0/6). The average duration of the operation in Group B was 79.38 ± 10.16 min (range, 60–90 min), which was significantly longer than in Group A (*p* < 0.001). No complications such as pin-site infection or iatrogenic nerve injury was found in this group.

**Table 1:** General descriptive data of the included Children.

	**Group A**	**Group B**	***P-*value**
No. of children	6	8	
Age at the presentation (yo)	5.00 ± 2.00	5.75 ± 3.69	0.197
Sex (male/female)	4/2	4/4	0.627
Side of injury (left/right)	3/3	3/5	1.000
Neurovascular involvement	0	0	
Fracture type			
Oblique	3	5	
Transverse	2	2	
Comminuted	1	1	
Interval from injury to surgery (D)	2.00 ± 0.63	2.25 ± 0.71	0.352
Pin configuration			
1 INRA, 2 LAT, 1 MED		1	
1 INRA, 1 LAT, 1 MED	6	0	
1 LAT, 1 MED	0	2	
2 LAT, 1 MED	0	5	
Need for open reduction	0/6	3/8	0.209
Surgery duration			
CR only	41.67 ± 7.53	79.38 ± 10.16	<0.001^*^
Re-operation	0/6	1/8	1.000

*yo, years old; D, days; INTRA, Intramedullary; LAT, lateral; MED, medial;*

* *significance*.

All the children in Group A had a successful CRPP procedure. The average Bauman angle was 69.00 ± 4.65° immediately after operation, 68.50 ± 6.02° at the time of pin removal, and 69.33 ± 5.79° at final radiographic follow-up. Similar results were observed among the five patients with successful CRPP in Group B (*n* = 5), with a Baumann angle of 67.60 ± 6.43° immediately after operation, 67.40 ± 5.03° at the time of pin removal, and 67.60 ± 4.51° at final radiographic follow-up. The lateral humeral-capitellar angle in Group A averaged 41.67 ± 5.50° on the immediate post-operative radiograph, 39.83 ± 4.45° at the time of pin removal, and 41.83 ± 5.08° at the last follow-up. In Group B, the lateral humeral-capitellar angle averaged 39.40 ± 7.13° on the immediate post-operative radiograph, 39.20 ± 4.49° at the time of pin removal, and 39.60 ± 5.18° at the last follow-up. No significant differences were observed at each check point, respectively (Table 2).

**Table 2:** Radiographic Outcomes of successful CR in two groups in two groups.

	**Group A (*n =* 6)**	**Group B (*n =* 5)**	***P-*value**
Baumann's angle			
Post-operative immediately	69.00 ± 4.65	67.60 ± 6.43	0.735
Pins removal	68.50 ± 6.02	67.40 ± 5.03	0.413
Last follow-up	69.33 ± 5.79	67.60 ± 4.51	0.73
Lateral humeralcapitellar angle			
Post-operative immediately	41.67 ± 5.50	39.40 ± 7.13	0.281
Pins removal	39.83 ± 4.45	39.20 ± 4.50	0.544
Last follow-up	41.83 ± 5.08	39.60 ± 5.18	0.774

At the last follow-up, the clinical outcomes among the cases with successful CRPP in the two groups were evaluated on the basis of the cosmetic and functional Flynn criteria. The average flexion of children in Group A (*N* = 6) was 138.33 ± 4.89°, the average extension was 1.33 ± 1.51°, and the mean ROM was 139.67 ± 4.93°. The average flexion of children with successful CRPP in Group B (*N* = 5) was 136.00 ± 4.53°, the average extension was 0.04 ± 2.07°, and the mean ROM was 136.40 ± 5.64°. The carrying angles of both injured and unaffected elbows in each group were measured. The mean carrying angle loss was 0.83 ± 1.72° in group A and 2.60 ± 1.14° in group B, respectively (Table 3). The clinical outcomes were classified as excellent, good, fair, or poor in accordance with the Flynn criteria. At last follow-up, although there were some loss of function and carrying angle in affected elbows when compared to the contralateral elbows, all the children in Groups A and B obtained excellent or good results. No patient was given a fair or poor grade either in the functional and cosmetic evaluation in either group.

**Table 3:** Clinical outcomes of successful CR in two groups.

	**Group A (*n =* 6)**	**Group B (*n =* 5)**	***P-*value**
Flexion of the FE	138.33 ± 4.89	136.00 ± 4.53	0.763
Flexion of the CE	141.17 ± 4.17	141.00 ± 5.70	0.828
***P-*****value**	0.035^*^	<0.001^*^	
Extension of the FE	1.33 ± 1.51	0.40 ± 2.07	0.398
Extension of the CE	3.00 ± 1.67	3.00 ± 2.35	0.152
***P-*****value**	<0.001^*^	<0.001^*^	
ROM of the FE	139.67 ± 4.93	136.40 ± 5.64	0.796
ROM of the CE	144.17 ± 5.64	144.00 ± 6.00	0.941
***P-*****value**	<0.001^*^	<0.001^*^	
Carrying angle of the FE	7.00 ± 1.26	6.60 ± 3.13	0.049
Carrying angle of the CE	7.83 ± 2.64	9.20 ± 2.77	0.952
***P-*****value**	0.008^*^	<0.001^*^	
Flynn's criteria			
Functional, loss of ROM (°)			
Excellent (0–5)	4	1	
Good (5–10)	2	4	
Fair (10–15)	0	0	
Poor (>15)	0	0	
Cosmetic, difference in CA (°)			
Excellent (0–5)	6	5	
Good (5–10)	0	0	
Fair (10–15)	0	0	
Poor (>15)	0	0	

*FE, fracture elbow; CE, contralateral elbow; ROM, range of motion; CA, carrying angle;*

* *significance*.

## Discussion

MDJ fracture is a rare elbow fracture occurring in children. Because of the specific site where the fracture line is located, the fracture possesses some intrinsic unstable characteristics. Compared to humeral supracondylar fractures, the area of the MDJ fracture section is smaller, and the periosteum in this region is vulnerable (8, 9). As a result, MDJ fractures may lead to some inevitable issues that are uncommonly encountered in treating typical supracondylar fractures. This study introduced a novel surgery technique for treatment of humeral MDJ fractures. The satisfactory outcomes may enhance the diagnostic awareness and optimize technical strategies for MDJ fractures.

In practice, one of the most problematic factors is that it is difficult to maintain reduction of the MDJ fracture. Displacement again after initial reduction is not uncommon, even with a cautious position shift. Anatomically, the cross-sectional area of the bone in the MDJ site is much smaller than the supracondyle of the humerus so that there is less contact surface to hold the reduction; as a result, MDJ fractures are much more unstable than supracondylar fractures. Mechanically, the MDJ fracture is more proximally located, and the increased length of lever arm makes the reduction unstable so that any small force may lead to re-displacement. Technically, the thin periosteum around the MDJ region is frail, and it is not likely to provide a competent hinge for manual reduction. The MDJ fracture is somewhat similar to the type IV supracondylar fracture, which is characterized by an incompetent periosteal hinge circumferentially and defined by instability in both flexion and extension (18, 19). In view of these characteristics, the optimum reduction maneuver in MDJ fracture is different from that used in the classical supracondylar fracture. In the present study, during manual correction under fluoroscopy, the assistant must maintain the reduction by holding the separated fracture ends constantly, and we obtained the different views of the reduction by rotating the C-arm instead of rotating the patient's arm. Both of these are beneficial in keeping the reduction stable and avoiding re-displacement. Moreover, a longitudinal intramedullary K-wire was first inserted to provide axial stabilization of the distal fracture fragment. In fact, the intramedullary K-wire was unlikely to guarantee a rigid fixation of the fracture. However, because of this axial stabilization, the distal fracture fragment could be further rotated by pronation or supination of the forearm, which was used to reduce rotational displacement in some cases.

In addition, the conventional pinning procedure utilized in supracondylar fractures after fracture reduction is not completely appropriate for the treatment of MDJ fractures. A number of studies have reported unsatisfactory outcomes in MDJ fractures using K-wires fixation (9, 15). Fayssoux et al. (9) treated MDJ fractures in the same manner as supracondylar humerus fractures. The MDJ fractures were more problematic because of loss of fixation and significantly increased operative time when compared to supracondylar humerus fractures (9). In the present study, we reviewed our MDJ fractures treated by the conventional CRPP methods that are utilized in treating supracondylar fractures and found that the need for open procedures was as high as 37.5% (3/8). Because of the higher fracture lines in the MDJ fracture, the angle that the pin must make to cross the fracture is so acute that the tip of the pin ends up being oriented almost parallel to the inner cortical surface of the opposite cortex, resulting in difficulty in acquiring cortical purchase (20). This is the main reason for the difficulty in fixation and the post-operative loss of fixation. According to the traditional procedure in treating humeral supracondylar fractures, pin placement is followed by reduction. However, when treating unstable MDJ fractures, the stress of the pins on the distal fragments during drilling may cause re-displacement of the reduced fracture. Even with sustained stabilization of the fracture by an assistant, the difficulty and time required for accurate pinning to secure opposite cortical purchase may cause the effort to fail. Instead, inserting the intramedullary K-wire as used in the present study makes it possible to ensure proper stabilization of the fracture and easy placement. The intramedullary K-wire insertion gives the MDJ fracture a great degree of fixation that transforms the multidirectional instability of the fracture into a merely rotational instability. With the axial stability provided by the intramedullary pin, the insertion of cross-pins becomes effortless. We believe that a benefit of the surgical technique described here is that an open reduction can be avoided. All of the six MDJ fracture cases had successful CRPP, and none of them required any further surgery. Furthermore, surgery duration is significantly shorter than that in children treated with the conventional technique.

In the present study, we selected cross-pin fixation followed by intramedullary pin insertion in all children because of the multidirectional unstable nature of MDJ fractures. Although the relative risk of iatrogenic ulnar nerve injury has been reported, it has been suggested that cross-pin configuration is associated with greater torsional stability than that of lateral pins (21, 22). Medial pin placement has been routinely used in our institution in treating distal humeral fractures. Very few iatrogenic ulnar nerve injuries have been observed (23). When inserting the medial pin, the elbow was kept in extension so that the ulnar nerve shifted posteriorly, and the pin was inserted from the anterior portion of the medial epicondyle; the orientation of pin track was from the anteromedial to posterolateral aspect of the distal humerus. With this method, there was no ulnar nerve irritation in any case.

Sen et al. reported five children with MDJ who were successfully treated by closed reduction and plaster of Paris cast under sedation (14). On the contrary, Cvitanich and Hoffman reported a high rate of failure of conservative treatment for MDJ fracture, in which three of four patients managed conservatively had a poor result with varus malunion (24). In the present study, all the MDJ fractures were displaced, and because of the unstable characteristics of this kind of fracture, the risk of re-displacement during casting cannot be ignored. In addition, percutaneous pinning is able to ensure fixation without injury. As a result, all the children with MDJ in the present study were treated with closed reduction and additional percutaneous pinning.

The use of elastic intramedullary nails has been reported to be a safe and efficient method for treating displaced long-bone fractures (25, 26). Some studies also reported the utilization of this technique in treating the MDJ fractures of the humerus (27, 28). However, we are uncertain about appropriateness of this technique in the treatment of this kind of atypical humeral fracture, which may be problematic because of the relatively short length of the distal fragment as well as the multidirectional instability of MDJ fractures. The maneuver of advancing the nail by tapping to cross the fracture line leads to the re-displacement of the initial reduced fracture. In addition, the skin and subcutaneous tissue involvement caused by intramedullary nails at their insertion site or the secondary surgery for removal of the nails should also be taken into consideration. A previous study by Marengo et al. reported using an elastic intramedullary nail technique to treat MDJ fractures; however, this study included some cases of humeral diaphyseal fracture, which is not the standard MDJ fracture (28).

There are limitations to the current study. This was a retrospective evaluation of surgically treated MDJ fractures in only a single center. The relatively small sample size limits statistical power, although results are similar to those in other published studies. Despite these limitations, the study demonstrated good functional and radiographic outcomes for humeral MDJ fractures in children treated with the novel technique.

In summary, the novel technique introduced in the present study can provide a credible alternative for the treatment of displaced MDJ fractures. In comparison with the conventional CRPP procedure, which is based on the treatment of supracondylar fractures, satisfactory clinical and radiographic outcomes can be achieved with shorter surgical duration and easier operation by using the novel technique. However, more cases and long-term follow-up are necessary to further validate this technique.

## Data Availability Statement

The raw data supporting the conclusions of this article will be made available by the authors, without undue reservation.

## Ethics Statement

The studies involving human participants were reviewed and approved by this was a retrospective study of patient data, and IRB approval was obtained from Children's Hospital of Chongqing Medical University (2020196). Written informed consent to participate in this study was provided by the participants' legal guardian/next of kin.

## Author Contributions

YZ, HZ, and GZ were involved in the conception and design of the project and participated the surgery implementation. HZ, GZ, YC, and LW collected and extracted the data. GZ, XL, and XQ conducted the analysis and data interpretation. YZ drafted the manuscript. YZ, HZ, XL, XQ, and YC made the critical revisions. All authors read, provided feedback, and approved the final manuscript.

## Conflict of Interest

The authors declare that the research was conducted in the absence of any commercial or financial relationships that could be construed as a potential conflict of interest.
